# Ubiquitin and Ubiquitin‐Like Modifications in Organelle Stress Signaling: Ub, Ub, Ub, Ub, Stayin’ Alive, Stayin’ Alive

**DOI:** 10.1002/bies.202400230

**Published:** 2024-11-26

**Authors:** Elodie Lafont, Eric Chevet

**Affiliations:** ^1^ INSERM UMR1242 Oncogenesis Stress Signaling Université de Rennes Rennes France

**Keywords:** adaptation, cell death, cellular stress, organelle, Ubiquitin, Ubiquitin‐like modifications

## Abstract

Due to various intracellular and external cues, cellular organelles are frequently stressed in both physiological and pathological conditions. Sensing these stresses initiates various signaling pathways which may lead to adaptation of the stressed cells or trigger its their death. At the unicellular level, this stress signaling involves a crosstalk between different organelles. At the multicellular level, such pathways can contribute to indicate the presence of a stressed cell to its neighboring cells. Here, we highlight the crucial and diverse roles played by Ubiquitin and Ubiquitin‐like modification in organelle stress signaling.

## Graphical abstract

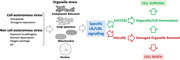

## 1 Introduction

**FIGURE 1:**
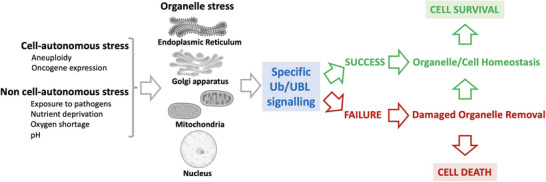
Schematic representation of organelle signaling and consequences.

Various types of intracellular or environmental insults can alter the integrity of organelles and their physiological functions. Ubiquitin (Ub) and UBL (Ubiquitin‐like) modifications are key actors in signaling these alterations to the rest of the cell, possibly leading to stress relief, and which may also include removal of parts of, or the whole, defective organelle if the stress cannot be resolved (Figure 1).

## 2 Broken: Ub/UBLs Signal Organelle Health Status Intra‐ and Inter‐Cellularly

Organelle stress, resulting in malfunctioning or damage of an organelle, can be relayed to the rest of the cell organelles and the surrounding cells in an Ub and UBL‐dependent manner. Almost all organelles in the cell can be stressed and signal to the rest of the cell.

In their recently published review, Endo et al. describe depletion of the K63‐ubiquitin targeting deubiquitinase USP8 as a driver of a phenomenon they coined endosome stress [1]. USP8 is a known regulator of plasma membrane receptor fate upon endocytosis. Indeed, USP8 can on the one hand mediate the removal of K63‐ubiquitin from endocytosed receptors such as EGFR and therefore promote their recycling to the plasma membrane. On the other hand, USP8 removes K48‐ubiquitin from components (HRS and STAM1/2) of the ESCRT‐0 complex, stabilizing these and therefore promoting the sorting of endocytosed plasma membrane receptors to lysosomes. Thus, the net result of USP8 engagement is clearing early endosomes of ubiquitinated receptors. Endo et al. report that genetic depletion of USP8 not only results in accumulation of ubiquitinated plasma membrane receptors at early endosomes as would be expected but also that the resulting accumulation of K63 ubiquitin chains at their surface recruits and activates TABs/TAK1/NF‐κB and p62/Keap1/Nrf2‐dependent signaling, promoting expression of immune‐related genes (such as CCL5, SOD2). Accordingly, conditioned media from USP8‐depleted HeLa cells promote STAT3 and ERK1/2 activation in parental HeLa cells, suggesting signaling to surrounding cells of the ongoing internal stress. Of note, USP8‐depletion–dependent accumulation of ubiquitin on endosomes does not induce their removal by autophagy as is observed for endosomes with loss of membrane integrity. Mutations of USP8 have been described in Cushing's disease, yet the relevance of endosomal stress in this disease remains unexplored. Given that oxidative stress can trigger depletion of USP8, it is also possible that endosomal stress might be triggered by external cues and relevant to other pathophysiological contexts.

Mitochondrial outer membrane permeabilization (MOMP) is a frequent event triggered by multiple types of apoptotic stress. If MOMP is major, it can result in the release of mitochondrial factors, such as cytochrome C, which will contribute to apoptotic signaling. MOMP can also be incomplete, and trigger non‐apoptotic outcomes. Permeabilized mitochondria can also release mtDNA, which will drive a Type I interferon response dependently on the cGAS/STING pathway. A recent study described that apoptotic MOMP can also directly drive inflammatory signaling depending on ubiquitin. Indeed, apoptotic MOMP results in K63‐ubiquitination of multiple proteins of the outer and inner mitochondrial membranes. This “ubiquitin coat” primarily recruits NEMO and promotes NF‐κB–dependent inflammatory signaling [2]. At later stages, however, mitochondria are degraded in a ubiquitin‐proteasome–dependent manner. This is reminiscent of earlier reports describing that the formation of an M1‐ubiquitin coat on vacuoles containing invading bacteria in host cells recruits NEMO which in turn drives NF‐κB activation and thus innate immune signaling to repress bacteria proliferation [3, 4]. Thus, ubiquitin accumulated on stressed organelles can initially serve to warn surrounding organelles and cells of the ongoing intracellular stress prior to triggering organelle removal.

An intriguing recent study revealed how features of the ubiquitinated substrates themselves can also time organelle stress signaling, and may condition its cellular outcomes. Indeed, upon mitochondrial protein stress import, the E3 ubiquitin ligase complex SIFI recognizes particular degrons which are shared by unimported mitochondrial proteins and by cDELE1, a sensor of mitochondria import stress as well as the Integrated Stress Response (ISR) kinase HRI, which represses overall protein synthesis [5]. The presence of such converging degrons which can compete for SIFI binding would therefore represent means to allow that ISR is engaged as long as the mitochondrial import stress lasts but not beyond.

Recent publications point toward the role of UFM1, a UBL, in multiple aspects of endoplasmic reticulum (ER) proteostasis maintenance, including ER‐phagy, ER‐associated protein degradation, and ribosome quality control, as reviewed in [6], UFM1 is for instance involved in the quality control of ribosomes that stall during the co‐translational translocation of proteins in the ER. UFMylation is thought to fulfill several functions in this process. Indeed, UFMylation of the 60S ribosomal protein RPL26 can promote the release of stalled, as well as normally terminated [7, 8], ribosomes from ER translocons, allowing the rescue and recycling of these subunits. While the full mechanism involved is not entirely clear, UFMylation also promotes the proteosomal degradation of aberrant nascent peptides, which are marked for degradation by the E3 ligase LTN1 recruited at stalled ribosomes. Hence, UFMylation allows for seamless stress‐solving in this context.

## 3 Good Riddance: Ub/UBL Roles in Organelle Removal (Organellophagy)

When organelle stress cannot be resolved, Ub and UBL modifications can act as “eat‐me” signals for damaged organelles, as well as promote the expansion of phagophores to engulf damaged organelles in various forms of organelle selective autophagy, also called organellophagy. The most studied example for these functions depends on ubiquitin and UBLs of the ATG8 family in mitophagy, a form of selective autophagy by which damaged mitochondria are removed through engulfment in autophagosomes and delivery to lysosomes. As such, upon sensing mitochondrial alterations, the kinase PINK1 is stabilized, tethered at the mitochondrial outer membrane (MOM), and activated to initiate the phosphorylation of pre‐existing ubiquitinated MOM proteins. Phospho‐ubiquitin then promotes the recruitment of the E3 ubiquitin ligase Parkin, which is further activated by PINK1‐mediated phosphorylation. Activated Parkin in turn ubiquitinates multiple MOM and MOM‐associated proteins (e.g., TOMM20, MFN1, CYB5R3, and VDAC1/2/3). These mono‐ and poly‐ubiquitinated substrates form a “ubiquitin coat” which is recognized by various ubiquitinated cargo receptors to trigger autophagosome assembly [9]. Receptors for ubiquitinated cargoes, such as optineurin, TAX1BP1, CALCOCO2, NBR1, and SQSTM1, display various selectivity and redundancy for subtypes of organellophagy. Structurally, they possess the ability to bind to ubiquitin and to sequentially bind FIP200, a component of the autophagic ULK complex, and ATG8 family members conjugated to phospholipids of the expanding phagophore [9]. Noteworthy, modification of phospholipids by ATG8 members is not only key in organellophagy because of ATG8 role in linking the phagophore to cargo receptors, but also because they influence the phagophore membrane curvature properties, as reviewed in [10].

Akin to the key steps described for mitophagy, xenophagy also relies on the generation of a ubiquitin coat. Xenophagy has been mostly studied for *Salmonella Typhimurium*. Upon infection, rupture of bacteria‐containing vacuoles in the host cell recruits galectin‐8 and triggers the formation of a ubiquitin coat, both contributing to the recruitment of autophagy machinery through cargo receptors (CALCOCO2, OPTN, and SQSTM1). Not only vacuole proteins but also the *Salmonella* membrane component lipopolysaccharide itself is ubiquitinated in this process [11]. Of note, an additional example of ubiquitination of lipid as being key in organelle stress signaling was also demonstrated for eukaryotic lipids one year later, as phosphoethanolamine has also been shown to be ubiquitinated upon starvation and lysosomal damage. LPS ubiquitination is catalyzed by the E3 ligase RNF213 and allows the recruitment of the E3 ligase LUBAC. LUBAC, by building multiple linear ubiquitin chains, further recruits autophagy cargo receptors, in addition to recruiting NEMO as mentioned earlier [3, 4], and can therefore contribute to xenophagy induction.

As mentioned earlier, UFMylation is key in taking charge of ER‐stalled ribosomes, driving detachment of stalled ribosomes, and ubiquitination of aberrant nascent peptides. Alternatively, and maybe as a result of overwhelming of the LTN1‐dependent ribosomal quality control, UFMylation of RPL26 and RPN1, a subunit of the translocon‐associated OST complex, can also promote the recruitment of phagophores through CDK5RAP3. CDK5RAP3, which is a component of the UFMylation E3 ligase complex, here acts as an autophagy receptor, recognizing both UFMylated proteins and LC3 at the expanding phagophore membrane, thus promoting degradation of portions of the ER displaying irremediably stalled ribosomes. Of note, the expression of all the components of the UFMylation machinery can be induced by the ER stress sensor IRE1 and is therefore part of the multiple mechanisms by which the UPR controls ER proteostasis. [12]

The removal of damaged organelles is by itself an adaptive response to protect the cell, however, if the damage is extensive, organelle removal cannot be sufficient to ensure organelle homeostasis and subsequently may lead to cell death.

## 4 Message in a Bottle: Ub/UBLs as Signaling Platforms on Organelles for Decoding Extracellular (and intracellular) Stress Cues

Beyond their role in signaling organelle stress, ubiquitin and UBL assembled on various cargos and organelles can also act as integrators and regulators of various stress signals. The role of ubiquitin accumulation in controlling the fate (degradation over recycling) of EGF‐stimulated EGFR has for instance long been described. More recently, engagement of organelle‐dependent signaling has been highlighted upon TNF stimulation. Engagement of TNFR1 by TNF triggers the formation of a TNFR1‐associated complex comprising several E3 ligases (cIAP1/2 and LUBAC) which ubiquitinate multiple components of the complex (e.g., RIPK1, TRADD) leading to the recruitment of the TABs/TAK1 and NEMO/IKKα/IKKβ complexes driving activation of NF‐κB signaling and repression of cell death. Beyond the well‐described formation of this TNFR1‐associated protein complex, optimal transmission of NF‐κB signaling upon TNF could in fact also require the engagement of multiple organelles, an intriguing phenomenon still understudied. Indeed, ubiquitinated RIPK1 has been shown to accumulate at the cytosolic surface of the ER upon TNF engagement dependently on the presence of the ER‐localized and K63‐ubiquitin binding protein MTDH [13]. This accumulation of ubiquitinated RIPK1 at the surface of the ER has been suggested to promote the formation of a NF‐κB‐activating signalosome. Whether this accumulation of ubiquitinated RIPK1 at the ER results from particular membrane dynamic events upon TNF stimulation (i.e., pre‐existing or de novo formation of ER‐plasma membrane or ER‐endosomes contact sites), and if such events depend on Ub/UBL, remains to be addressed. Of note, the same study also highlighted that accumulation of ubiquitinated NF‐κB signaling components at the ER is a shared pattern which also occurs upon engagement of multiple immune receptors (CD40, TCR, NOD2, and various TLRs) and contributes to their signaling.

Winklhofer and team recently put forward an additional layer of organelle and UB‐dependent regulation of TNF signaling. [14] They described that TNF stimulation triggers the recruitment of LUBAC and linear ubiquitin chain formation at the mitochondria outer membrane. This promotes PINK1 stabilization but does not result in increased mitophagy. Instead, PINK1, by phosphorylating ubiquitin, represses the activity of the M1‐degrading deubiquitinase OTULIN, which the authors suggest to contribute to mitochondria‐localized activation of NEMO‐dependent NF‐κB signaling. Very intriguingly, the authors also describe that the phosphorylated NF‐κB subunit p65 is associated with mitochondrial membranes upon TNF stimulation. Furthermore, they describe that TNF stimulation increases contact sites between mitochondria and nucleus in a linear ubiquitination‐dependent manner and hypothesize that this phenomenon could contribute to increased delivery of activated NF‐κB to the nuclei, and thus inflammatory signaling.

## 5 Conclusion

Taken together, the above‐mentioned examples highlight several patterns involved in the spatio‐temporal regulation of organelle stress signaling by ubiquitin and UBLs. As such Ub/UBL‐dependent organelle signaling might constitute a specific code which could define stress signaling duration, the nature of downstream signaling pathways activated and in turn the cellular fate of the organelle which by extent might also condition cell's life and death decisions. Beyond this impact of Ub/UBL on organelle stress signaling, recent studies have discovered that Ub/UBL‐mediated signaling could apply to membrane‐less structures [15]. Indeed, the UBL URM1 self‐associates and phase separates along with its E1 enzyme UBA4, which depends on the acidification of the cellular milieu. This promotes the URMylation of multiple proteins which co‐partition into perinucleolar condensates and stress granules through covalent and non‐covalent interactions with URM1 to repress global translation. In conclusion, the Ub/UBL‐dependent organelle stress signaling is likely to represent a universal mechanism to control cell adaptation which may constitute an extensive and diverse coding post‐translational modification (PTM) system, which due to its diversity could be far more versatile than the most described PTMs so far.

## Author Contributions

EL and EC wrote the manuscript.

## Conflict of Interest Statement

The authors declare no conflicts of interest.

## Data Availability

Data sharing is not applicable to this article as no new data were created or analyzed in this study.
